# Reliable evaluation of tumor-infiltrating lymphocytes in pancreatic cancer tissue biopsies

**DOI:** 10.18632/oncotarget.26646

**Published:** 2019-02-01

**Authors:** Yoshinori Ino, Seiji Oguro, Rie Yamazaki-Itoh, Shutaro Hori, Kazuaki Shimada, Nobuyoshi Hiraoka

**Affiliations:** ^1^ Division of Molecular Pathology, National Cancer Center Research Institute, Tokyo, Japan; ^2^ Department of Analytical Pathology, National Cancer Center Research Institute, Tokyo, Japan; ^3^ Division of Pathology and Clinical Laboratories, National Cancer Center Hospital, Tokyo, Japan; ^4^ Hepatobiliary Pancreas Surgery Division, National Cancer Center Hospital, Tokyo, Japan

**Keywords:** pancreatic cancer, biopsy, tumor-infiltrating lymphocytes, intraclass correlation coefficient, quantitative RT-PCR

## Abstract

Tumor-infiltrating lymphocytes (TILs) represent cancer microenvironment. We previously reported TILs was prognosticators in pancreatic ductal adenocarcinoma (PDAC) patients by immunohistochemically measuring them in surgically-resected tissues. The aim of this study was to assess how best to evaluate TILs in PDAC tissue biopsies. First, we showed expression of *CD3*, *CD4*, or *CD8* genes in PDAC tissue measured by quantitative RT-PCR (RT-qPCR) was prognostic using 241 surgically-resected specimens. We assessed whether the TILs in biopsied tissues can be effectively evaluated by comparing between immunohistochemistry and RT-qPCR. As a study model, we sampled twenty biopsies from surgically-resected PDAC specimen (*n* = 17). We investigated the variation levels of TILs in the different biopsies from the same specimen and evaluated using the intraclass correlation coefficient (ICC). The ICC value was 0.58 for *CD3*, 0.61 for *CD4*, and 0.46 for *CD8*, respectively; these ICC values meant correlations of “moderate” to “substantial” levels. To reach “near perfect”, 3, 3, and 5 times biopsies were necessary for *CD3, CD4*, and *CD8*, respectively. When ICC values of immunolabeled TILs were of “low”, ≥6 times biopsies were necessary to reach “moderate” levels. We found that TILs measured by RT-qPCR and repeated sampling increased reliability in TILs detected from biopsied PDAC tissues.

## INTRODUCTION

Pancreatic ductal adenocarcinoma (PDAC), one of the most common and devastating cancers, has a 5-year survival rate of only 8% [1]. Given that PDAC exhibits aggressive growth and early metastatic dissemination and that no clinically informative early diagnostic symptoms and biomarkers are available for PDAC, most patients are identified too late for treatment by curative surgical resection. A thorough understanding of PDAC is necessary to develop new treatment modalities for patients with unresectable PDAC.

Given that tumor-infiltrating lymphocytes (TILs) represent the host antitumor immune response in several types of cancer [2], TILs can be prognosticators. We and others have reported that the presence of tumor-infiltrating CD4^+^ or CD8^+^ T cells and the ratio of tumor-infiltrating FOXP3^+^ Tregs to CD4^+^ T cells were favorable and unfavorable prognosticators, respectively, in patients with PDAC [3–5]. In these studies, TILs were analyzed using surgically resected PDAC tissues. However, specimen biopsies are the only PDAC tissue samples available from most patients, as more than 80% of them have unresectable PDAC upon initial diagnosis.

Recently, the development of new cancer immunotherapies that stimulate the host immune response again has resulted in sustained tumor elimination. Treatment with nivolumab or pembrolizumab, antibodies against PD-1, has dramatically improved the overall survival of patients with malignant tumors with poor prognoses [6, 7]. However, immune checkpoint inhibitors are not always effective and only patients with specific types of cancer or under limited conditions have benefitted. PDAC is one of the cancers mounting the poorest response to such treatments [8, 9]. Many researchers have studied to identify potential predictive markers for these new immunotherapies by investigating the tumor microenvironment including tumor-infiltrating immune cells. However, we still do not have context for the evaluation of TILs from the biopsies of PDAC tissues.

As personalized medicine progresses, several companion diagnostics have been developed for selecting appropriate patients for treatment. Tissue biopsies are small, with a very limited area yielding inadequate information on the position of the cancer tissue. Therefore, the evaluation criteria for tissue biopsies are often different from those for large-sized surgically resected tissue in companion diagnostics, i.e., HER2 testing for gastroesophageal adenocarcinoma [10]. TILs represents a promising candidate marker to assess host immune status for immunotherapy. Evaluation of TILs in tissue biopsies has been studied in breast cancer [11], although few if any studies have been done for other cancers including PDAC. Immune checkpoint therapy is effective in very limited numbers of PDAC patients [8, 9], and currently combination therapies with immune checkpoint therapy are also examined. It is more effective to perform biomarker analysis to determine the immune status for selecting patients suitable for therapy.

The aim of this study was to investigate an effective method for the evaluation of TILs in PDAC tissue biopsies. There are two possible methods for measuring TILs in PDAC tissue - immunohistochemistry and quantitative reverse transcription PCR (RT-qPCR). To determine whether RT-qPCR could be used to assess the tumor immune microenvironment, we measured TIL-related gene expression levels by RT-qPCR in 241 surgically-resected PDAC tissues and evaluated their usefulness as prognostic indicators. We went on to assess whether the TILs in the small tissue biopsies can be effectively evaluated by comparing the two methods under consideration. In total, we performed 20 biopsies on a freshly resected PDAC surgical specimen and measured the TILs in each biopsy tissue by RT-qPCR as well as immunohistochemistry. We then investigated the level of variation of each TIL variable in the different tissue biopsies from the same PDAC specimen. Intrarater reliability was assessed statistically using the intraclass correlation coefficient (ICC). We further examined the number of biopsies necessary to assess TILs with reproducible results.

## RESULTS

### Prognostic significance of gene expression levels of *CD3*, *CD4*, *CD8*, and *FOXP3* in PDAC tissues

**Figure 1:**
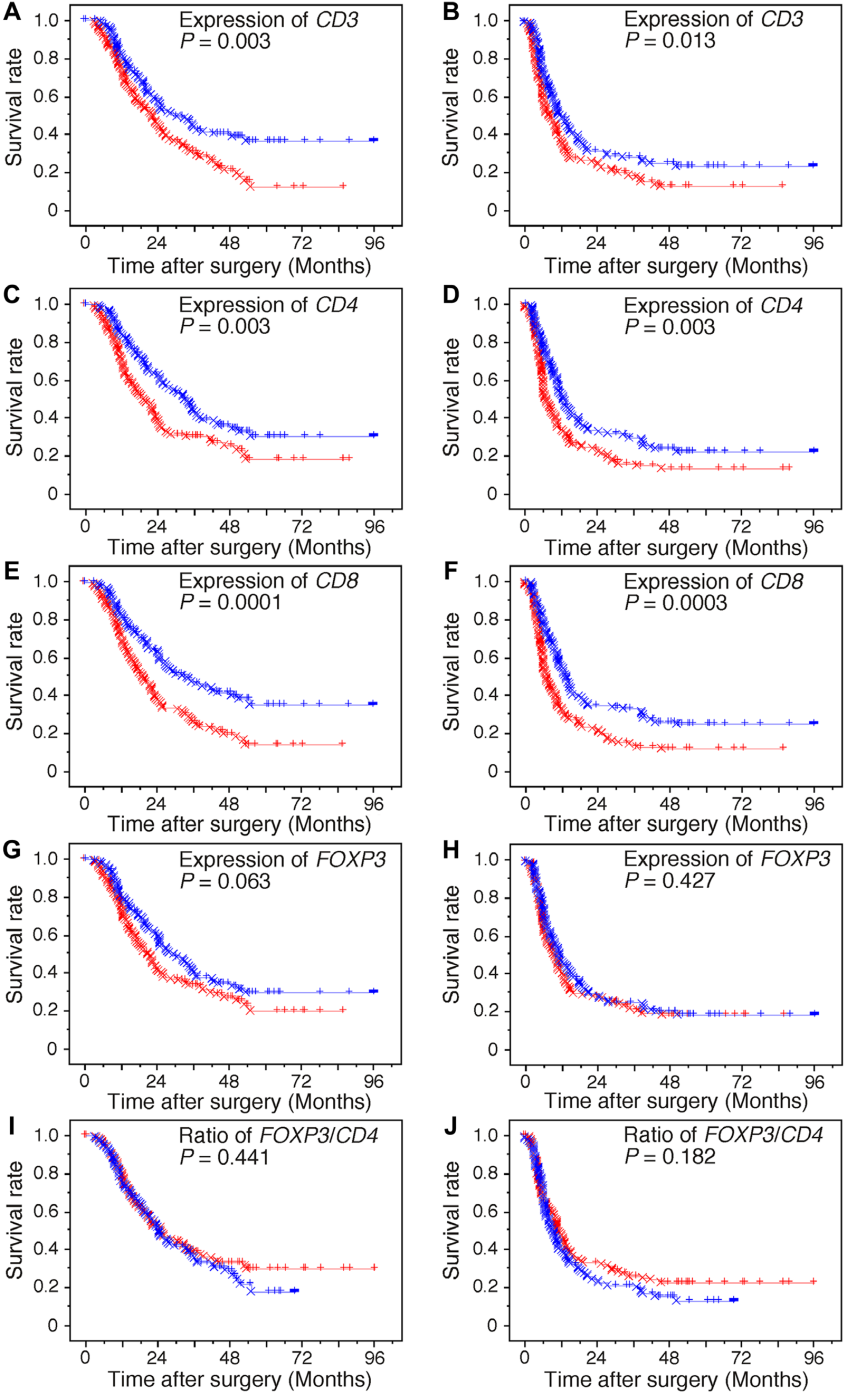
Kaplan–Meier survival curves Kaplan–Meier survival curves showing comparison of overall survival (**A, C, E, G,** and **I**) and of disease-free survival (**B, D, F, H,** and **J**) between higher (blue) and lower (red) expression levels of genes and the ratio of genes. The “circle” and “x” represent failure and censoring, respectively.

We showed previously that higher levels of tumor-infiltrating CD4^+^ T cells and tumor-infiltrating CD8^+^ T cells were significantly associated with longer survival in patients with PDAC, whereas higher levels of the ratio of tumor-infiltrating FOXP3^+^ Tregs to CD4^+^ T cells were significantly associated with shorter survival [4, 5]. Along with the analysis of immunolabeled lymphocytes by immunohistochemistry, we performed survival analysis by assessing the gene expression levels of *CD3, CD4, CD8*, and *FOXP3* in PDAC tissues, as these genes are nearly exclusively expressed in CD3^+^ T, CD4^+^ T, CD8^+^ T, and FOXP3^+^ Treg cells, respectively. Kaplan–Meier survival analysis revealed that a higher expression of *CD3, CD4*, and *CD8* was significantly associated with both longer OS and DFS in PDAC patients (Figure 1). No significant differences were found in *FOXP3* and the ratio of *FOXP3/CD4*. Median survival time, one-, two-, and five-year survival rate (Supplementary Table 1), and Cox proportional analysis of the groups categorized by each of the tumor-infiltrating cell parameters and conventional clinicopathological variables are summarized in Table 1. When the variables found to be significant by univariate analysis were subjected to multivariate analysis, higher T and M factors and lymphatic invasion, positive tumor margin, and lower expression of *CD8* were closely associated with shorter OS. In addition, higher age, T factor, M factor, and venous invasion, positive tumor margin, and lower expression of *CD8* were significantly associated with shorter DFS.

Table 1Univariate and multivariate analyses of prognostic factors associated with overall survival in patients with ductal carcinoma of the pancreas

**Table:** 

Variables	Univariate analysis		Multivariate analysis	
	HR (95% CI)	*P* value	HR (95% CI)	*P* value
Age (<60 years/≥60 years)	1.374 (1.002–1.884)	**0.048**		
Gender (female/male)	0.958 (0.694–1.322)	0.794		
Pathologic tumor status (T1+T2/T3)	3.876 (1.589–9.459)	**0.003**	2.891 (1.212–7.092)	**0.020**
Pathologic node status (N0/N1)	2.004 (1.336–3.005)	**0.0008**		
Pathologic metastasis status (M0/M1)	2.047 (1.311–3.195)	**0.002**	1.629 (1.040–2.778)	**0.033**
Histological grade (G1/G2+G3)	1.488 (0.887–2.498)	0.132		
Tumor margin status (negative/positive)	1.887 (1.363–2.613)	**0.0001**	1.629 (1.040–2.553)	**0.002**
Nerve plexus invasion (absence/presence)^a^	1.774 (1.246–2.525)	**0.002**		
Lymphatic invasion (0, 1/2, 3)^a^	1.971 (1.340–2.899)	**0.0006**	1.768 (1.191–2.625)	**0.005**
Venous invasion (0, 1/2, 3)^a^	1.947 (1.305–2.907)	**0.001**		
Intrapancreatic neural invasion (0, 1/2, 3)^a^	1.998 (1.379–2.894)	**0.0003**		
Adjuvant chemotherapy (positive/negative)	0.981 (0.686–1.402)	0.915		
Expression of *CD3* (high/low)	1.629 (1.184–2.242)	**0.003**		
Expression of *CD4* (high/low)	1.606 (1.170–2.206)	**0.003**		
Expression of *CD8* (high/low)	1.847 (1.340–2.544)	**0.0002**	2.007 (1.450–2.778)	**<0.0001**
Expression of *FOXP3* (high/low)	1.348 (0.983–1.848)	0.064		
Ratio of *FOXP3/CD4* (high/low)	0.883 (0.644–1.212)	0.442

**Table:** Univariate and multivariate analyses of prognostic factors associated with disease-free survival in patients with ductal carcinoma of the pancreas

Variables	Univariate analysis		Multivariate analysis	
	HR (95% CI)	*P* value	HR (95% CI)	*P* value
Age (<60 years/≥60 years)	1.477 (1.106–1.973)	**0.008**	1.367 (1.014–1.841)	**0.040**
Gender (female/male)	0.957 (0.712–1.285)	0.769		
Pathologic tumor status (T1+T2/T3)	3.492 (1.638–7.446)	**0.001**	2.705 (1.258–5.816)	**0.011**
Pathologic node status (N0/N1)	2.219 (1.512–3.258)	**<0.0001**		
Pathologic metastasis status (M0/M1)	2.437 (1.621–3.662)	**<0.0001**	2.077 (1.364–3.163)	**0.0007**
Histological grade (G1/G2+G3)	1.879 (1.155–3.059)	**0.011**		
Tumor margin status (negative/positive)	1.881 (1.392–2.543)	**<0.0001**	1.479 (1.077–2.030)	**0.016**
Nerve plexus invasion (absence/presence)^a^	1.762 (1.279–2.427)	**0.0005**		
Lymphatic invasion (0, 1/2, 3)^a^	1.804 (1.269–2.564)	**0.001**		
Venous invasion (0, 1/2, 3)^a^	2.254 (1.547–3.284)	**<0.0001**	1.903 (1.294–2.797)	**0.001**
Intrapancreatic neural invasion (0, 1/2, 3)^a^	2.042 (1.459–2.859)	**<0.0001**		
Adjuvant chemotherapy (positive/negative)	1.174 (0.834–1.652)	0.358		
Expression of *CD3* (high/low)	1.441 (1.078–1.926)	**0.014**		
Expression of *CD4* (high/low)	1.552 (1.160–2.075)	**0.003**		
Expression of *CD8* (high/low)	1.706 (1.274–2.285)	**0.0003**	1.743 (1.294–2.346)	**0.0003**
Expression of *FOXP3* (high/low)	1.124 (0.842–1.502)	0.427		
Ratio of *FOXP3/CD4* (high/low)	0.821 (0.614–1.098)	0.183		

^a^Classified according to the classification of pancreatic carcinoma of Japan Pancreas Society.

### Interrelationships between clinicopathological variables and the expression levels of *CD3, CD4, CD8*, and *FOXP3* in PDAC tissues

We analyzed the interrelationships between clinicopathological variables of PDAC and gene expression levels of *CD3, CD4, CD8*, and *FOXP3* in PDAC tissues (Table 2). Gene expression of *CD3, CD4, CD8*, and *FOXP3* showed significant and positive correlations with each other. *FOXP3/CD4* was significantly correlated with the expression of *FOXP3*. No significant correlation of these gene expression levels with conventional clinicopathological variables was found.

**Table 2:** Interrelationship between clinicopathological variables

Variables	gender	T	N	M	histology	ly^a^	v^a^	ne^a^	plx^a^	margin	chemotherapy	CD3	CD4	CD8	FOXP3	FOXP3/CD4
Age (≥60 years/ < 60 years)	NS	NS	0.038	NS	NS	0.005	NS	0.007	0.011	NS	NS	NS	NS	NS	NS	NS
Gender (male/ female)		NS	NS	NS	NS	NS	NS	NS	NS	NS	NS	NS	NS	NS	NS	NS
Pathologic tumor status (T1+T2/ T3)			<0.0001	NS	NS	0.008	NS	<0.0001	<0.0001	0.010	NS	NS	NS	NS	NS	NS
Pathologic node status (N0/ N1)				<0.0001	0.010	<0.0001	0.0002	0.010	0.001	<0.0001	NS	NS	NS	NS	NS	NS
Pathologic metastasis status (M0/ M1)					NS	NS	NS	NS	NS	0.012	NS	NS	NS	NS	NS	NS
Histological grade (G1/ G2, G3)						NS	0.001	NS	NS	NS	NS	NS	NS	NS	NS	NS
Lymphatic invasion (0, 1/ 2, 3)^a^							<0.0001	0.003	0.019	0.001	NS	NS	NS	NS	NS	NS
Venous invasion (0, 1/ 2, 3)^a^								0.0002	0.0006	0.003	NS	NS	NS	NS	NS	NS
Intrapancreatic neural invasion (0, 1/ 2, 3)^a^									<0.0001	<0.0001	NS	NS	NS	NS	NS	NS
Nerve plexus invasion (absence/ presence)^a^										<0.0001	NS	NS	NS	NS	NS	NS
Pathological margin status (negative/ positive)											NS	NS	NS	NS	NS	NS
Adjuvant chemotherapy (positive/ negative)												NS	NS	NS	NS	NS
Expression of *CD3* (low/ high)													<0.0001	<0.0001	<0.0001	NS
Expression of *CD4* (low/ high)														<0.0001	<0.0001	NS
Expression of *CD8* (low/ high)															<0.0001	NS
Expression of *FOXP3* (low/ high)																<0.0001
Ratio of *FOXP3/CD4* (low/ high)																

^a^Classified according to the classification of pancreatic carcinoma of Japan Pancreas Societ.

NS: not significantly correlated (*P* > 0.05), T: pathologic tumor status, N: pathologic node status, M: pathologica metastasis status, histology: histological grade, ly: lymphatic invasion, v: venous invasion, ne: intrapancreatic neural invasion, plx: nervve plexus invasion, margin: pathological margin status, chemotherapy: adjuvant chemotherapy, *CD3*: expression of *CD3 CD4*: expression of *CD4*, *CD8*: expression of *CD8*, *FOXP3*: expression of *FOXP3*, *FOXP3/CD4*: ratio of *FOXP3/CD4.*

### Intraclass correlation coefficient (ICC) of TILs measured as expression levels of *CD3, CD4, CD8*, and *FOXP3* in PDAC tissue biopsies

In the study model, we sampled twenty biopsy tissues (FNA biopsy) from a fresh surgical specimen resected from PDAC patients (*n* = 17); ten tissue biopsies were used for RT-qPCR and immunohistochemistry analyses, each. The ICC (r) for their variables was measured (Table 3). The ICC (r) was 0.58 for *CD3*, 0.61 for *CD4*, 0.46 for *CD8*, and 0.41 for *FOXP3* gene expression, respectively, and 0.42 for *FOXP3/CD4*. A *r*-value of 0.0–0.20 correlates with “slight,” 0.21–0.40 with “fair,” 0.41–0.60 with “moderate,” 0.61–0.80 with “substantial,” and 0.81 with “near perfect” [12]. The ICC values for gene expression were categorized from “moderate” to “substantial” levels (0.41–0.61).

**Table 3:** ICC and variance

Variables	ICC (1,1)			BMS	WMS	SEM
	*r*	95% CI	*P* value			
Expression of *CD3* gene	0.58	0.40 to 0.77	<0.001	6220	428	20.7
Expression of *CD4* gene	0.61	0.44 to 0.79	<0.001	579	35.0	5.91
Expression of *CD8* gene	0.46	0.29 to 0.68	<0.001	724	76.5	8.75
Expression of *FOXP3* gene	0.41	0.24 to 0.64	<0.001	339	42	6.5
Ratio of *FOXP3/CD4*	0.42	0.25 to 0.65	<0.001	906	109	10.5
CD3^+^ TILs	0.08	-0.03 to 0.30	0.08	2720	1690	41.1
CD4^+^ TILs	0.12	-0.00 to 0.36	0.03	1360	689	26.2
CD8^+^ TILs	0.02	-0.06 to 0.19	0.35	6410	5760	75.9
FOXP3^+^ TILs	0.22	0.07 to 0.48	<0.001	516	170	13.0
Ratio of FOXP3^+^/CD4^+^ TILs	0.15	0.01 to 0.42	0.01	1.83	0.82	0.91

Abbreviations: ICC: intraclass correlation coefficient, CI: confidence interval, BMS: between-targets mean square, WMS: within-target mean square, SEM: standard error, TILs: tumor-infiltrating lymphocytes.

**Figure 2:**
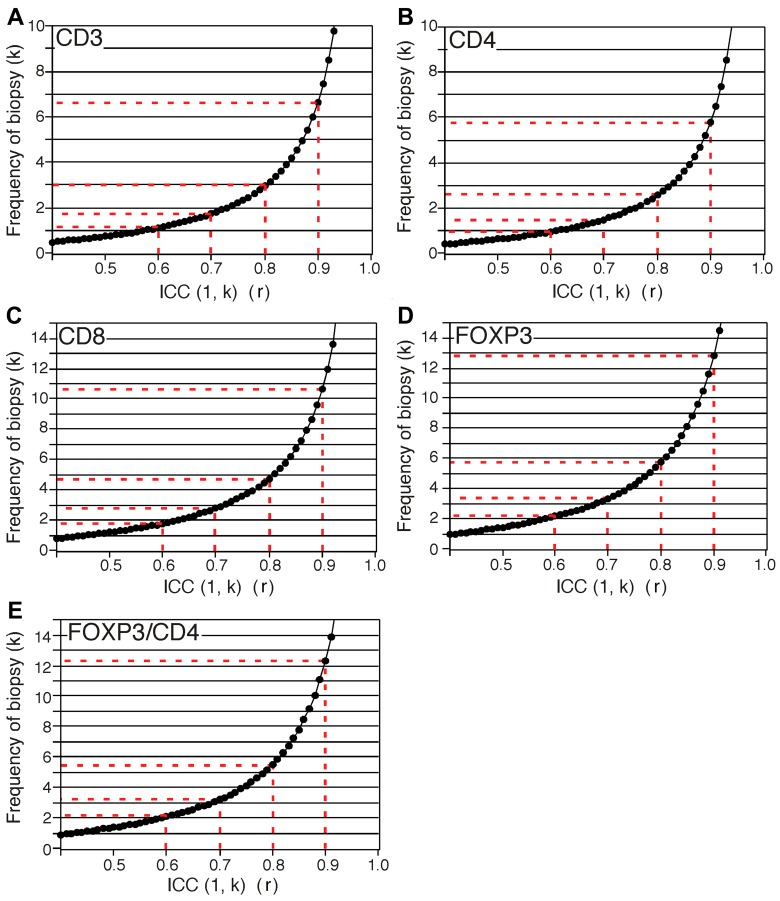
Predicted curves of ICC (1, k) for expression of *CD3* gene (**A**), *CD4* gene (**B**), *CD8* gene (**C**), and *FOXP3* gene (**D**) and ratio of *FOXP3* to *CD4* (**E**). x-axis and y-axis show ICC (1, k) values and frequency of the number of biopsy, respectively.

The ICC (1, k) of each variable was calculated and plotted (Figure 2). At the “near perfect” level (*r* > 0.8), at least 3, 3, 5, and 6 times the number of biopsies are necessary for *CD3, CD4, CD8*, and *FOXP3*, resepectively (Figure 2).

We compared gene expression levels between surgical specimens and biopsied tissue samples obtained from the same surgical specimen (Supplementary Figure 1). Gene expression levels in the surgically-resected tissues were placed almost always between lower and upper quartiles of the expression level in the corresponding biopsied tissue and majority were placed near the median. When the gene expression cut-off value for predicting outcome was set at the median of 241 cases of surgically-resected PDAC, both gene expression values in a given PDAC specimen were assigned to the same categories in all the cases and in all the genes tested. This suggested that the repeated biopsy confirmed the gene expression values seen in the surgically-resected tissue samples.

### ICC of TILs determined by immunohistochemistry in PDAC tissue biopsies

The ICC was 0.08 in CD3^+^ T cells, 0.12 in CD4^+^ T cells, 0.02 in CD8^+^ T cells, 0.22 in FOXP3^+^ Treg cells, and 0.15 in the ratio of FOXP3/CD4 (Table 3): these ICC values were categorized as “low” level (0.02–0.22). ≥6 times the number of biopsies are necessary to reach the “moderate” level (*r* > 0.4) (data not shown).

Tissue volume obtained by biopsy from the fresh resected PDAC specimen was very low (Supplementary Figure 2), similar to the Endoscopic Ultrasound (EUS)/Fine Needle Aspiration (FNA) (EUS-FNA) specimens collected from clinical PDAC tissue. PDAC tissue biopsies were highly fragmented and intermixed.

## DISCUSSION

In this study, we showed that higher expression levels of *CD3, CD4*, or *CD8* genes were significantly associated with both longer OS and DFS in PDAC patients according to Kaplan–Meier survival analysis. Higher expression levels of the *CD8* gene alone were significantly associated with both longer OS and DFS in multivariate survival analysis. These results suggest that the expression levels of *CD3, CD4*, or *CD8* genes in PDAC tissues could substitute for TILs determined by immunohistochemistry in surgical specimens, even though gene expression analysis is inferior to the variation in TILs determined by immunohistochemistry [3–5]. We also investigated the variation levels of each TIL variable in the different tissue biopsies from the same PDAC specimen. We showed that the ICC values of *CD3, CD4*, and *CD8* expression were categorized into “moderate” to “substantial” levels (0.41–0.61), while those of immunolabeled TILs were categorized into the “low” level (0.02–0.22). Furthermore, we predicted that in order to reach the “near perfect“ level (*r* = 0.8), at least 3 biopsies for *CD3*, 3 biopsies for *CD4*, 5 biopsies for *CD8*, and 6 biopsies for *FOXP3* are necessary (Figure 2), while ≥6 biopsies are necessary to reach the “moderate” level (*r* = 0.4) in immunolabeled TILs. These results recommend that TILs levels be assessed by the analysis of the expression levels of their related genes in PDAC tissue biopsies. They also suggest that the reliability of TILs data increases if PDAC tissues are biopsied and analyzed repeatedly. For reliable immunohistochemical analysis of TILs, it is necessary to perform repeated biopsies several times.

This study revealed that the TILs data determined by immunohistochemistry in PDAC tissue biopsies were not reproducible. PDAC tissue has generally desmoplastic stroma, making it difficult to collect a large quantity of tissues by aspiration biopsy, even under strong aspiration. In addition to the small amount of biopsied tissue, the tissue is highly fragmented and randomly intermixed. Furthermore, thinly-sliced tissue sections must be prepared by cutting FFPE blocks containing small tissue biopsies for immunohistochemistry. The chance of an uneven distribution of TILs throughout the specimen block is high. As a result, substantial variation is likely present in the data obtained between each tissue slide immunostained for TILs. However, the level of difficulty in collecting abundant tissue by biopsy is dependent on the tissue characteristics, which vary with the type of cancer.

We observed that immune cells are heterogeneously distributed within the tumor tissues and are sometimes infiltrated for reasons other than responding to cancer cells, such as ulceration and necrosis [5]. To represent the host antitumor immune reaction, TILs are often counted at hot spots (highly infiltrating area) and by avoiding the areas infiltrated for other apparent reasons (e.g., necrosis, ulceration). TILs determined by such methods are reproducible prognosticators in PDAC [5]. In contrast, the estimate of TILs by gene expression corresponds to an average of those in the tissue, given that total RNA is extracted from fresh frozen PDAC tissues. This method does not provide information on the location or position of the region of interest when counting the immune cells against the tumor or organ, which represents a substantial weakness in the method compared to immunohistochemistry. Nevertheless, some TIL variables were prognostic in univariate analysis but they were not independent prognosticators except for TILs estimated by the expression levels of the *CD8* gene. Although TILs estimated by gene expression have the advantage of reliability in small sample biopsies, they might remain subordinate to the TILs determined by immunohistochemistry in PDAC tissues surgically resected with respect to the positioning.

Our findings suggested that one-time biopsy does not provide reliable data in this study model. Sensitivity differs depending on target cell types, detection methods, and probably cancer/tissue types. To obtain reproducible results, repeated biopsy or the use of much larger-sized needles for biopsy are necessary to obtain PDAC tissue samples. PDAC tissue has a dense fibrous tissue that makes it tougher to collect large amount of tissue samples by aspiration biopsy. However, when cancer tissue is softer as in pancreatic neuroendocrine neoplasm, more tissue volume can be obtained through the same method.

The limitation of the present study was the use of only one study model for analyzing the evaluation of the tissue biopsies. Furthermore, interrater reliability was not investigated and the fact that it was a retrospective study meant that the possibility of selection bias could not be excluded.

In conclusion, we suggest that gene expression levels of *CD3, CD4*, and *CD8* in PDAC tissues are prognosticators for patients with PDAC. We determined that reliability increases in TILs detected in small biopsies of PDAC tissues, if TILs are estimated by the expression levels of their related genes in repeated biopsies.

## MATERIALS AND METHODS

### Study population

This study was approved by the Institutional Review Board of the National Cancer Center, Japan. Informed consent was obtained from all the participants involved in the study, and all clinical investigations were conducted in line with the principles of the Declaration of Helsinki. Clinical and pathological data and the specimens used for immunohistochemical analysis were obtained through a detailed retrospective review of the medical records of all 241 patients with PDAC who had undergone surgical resection between 2002 and 2008 at the National Cancer Center Hospital, Tokyo, and in whose case fresh frozen tissue was obtained from the resected specimen at surgery. None of the patients had received any therapy before surgery. All patients included in this study underwent macroscopic curative resection and all cases involved conventional ductal carcinomas; adenocarcinomas originating in intraductal papillary mucinous neoplasms or mucinous cystic neoplasms were excluded, as were secondary tumors and post-neoadjuvant cases. The clinicopathological characteristics of the patients are summarized in Table 4. The median follow-up period after surgery for all patients and for the living patients was 21.2 (3.1–96.3) and 44.2 (3.6–96.4) months, respectively. Each patient received follow-up care in the outpatient clinic every one to three months during the first postoperative year and every six to twelve months thereafter. Unless there was confirmation of disease relapse, patients underwent physical examination, laboratory tests, chest radiography, abdominal computed tomography, and/or ultrasonography. The tumor markers, carcinoembryonic antigen and carbohydrate antigen 19-9, were also measured until relapse. Recurrence was suspected when a new local or distant metastatic lesion was found on serial images and an increase in tumor-marker levels was recognized. When progression of the disease was confirmed by repeated imaging studies, the date of the first suspicious radiologic finding was used as the date of initial disease recurrence. At the census date (September 2011), we checked the survival status of the patients and found that 59 patients (24.5%) were alive, 165 (68.5%) had died of pancreatic cancer, and 17 (7.1%) had died of other causes. All M1 (based on TNM classification) patients had nodal metastasis around the abdominal aorta, without any other form of metastasis.

**Table 4:** Clinicopathological characters

Age, years		
	<65	107
	≥65	134
Gender		
	Male	144
	Female	97
Pathologic tumor status		
	T1a/1b/1c	1/0/17
	T2	133
	T3	90
	T4	0
Pathologic node status		
	N0	61
	N1	91
	N2	89
Pathologic metastasis status		
	M0	213
	M1	28
Stage		
	IA	13
	IB	34
	IIA	14
	IIB	89
	III	63
	IV	28
Tumor grade		
	Grade 1	32
	Grade 2	163
	Grade 3	46
Nerve plexus invasion^a^		
	Absence	86
	Presence	155
Lymphatic invasion^a^		
	0, 1	66
	2, 3	175
Venous invasion^a^		
	0, 1	62
	2, 3	179
Intrapancreatic neural invasion^a^		
	0, 1	80
	2, 3	161
Tumor margin status		
	Negative	160
	Positive	81
Adjuvant chemotherapy		
	Negative	184
	Positive	57
Total		241

^a^Classified according to the classification of pancreatic carcinoma of Japan Pancreas Society.

For the biopsy study, we collected samples prospectively from 19 patients who had undergone initial surgical resection for PDAC between November 2012 and June 2013 at the National Cancer Center Hospital, Tokyo, and whose fresh biopsy tissues were obtained from the resected specimen at the time of surgery. Seventeen of these cases were investigated, given that 20 biopsy samples per case were necessary for gene expression analysis and immunohistochemistry. The demographic information of the patients is shown in Supplementary Table 2.

### Biopsy

Fine-needle aspiration (FNA) biopsy procedures were performed using Sonopsy™-C1-21G (Hakko; Osaka, Japan), a biopsy needle used routinely for ultrasonography-guided FNA biopsy for clinical applications in our hospital. FNA was repeated 20 times on a fresh surgical specimen under macroscopic observation while checking for the presence of tumor mass by palpation. All biopsies were confirmed macroscopically to contain adequate material. The first collection of ten sample biopsies was immediately frozen in liquid nitrogen and stored at –80°C until analysis. The second collection of ten samples was fixed in 10% formalin for subsequent immunohistochemistry analysis and morphological examination.

### Pathological examination

All of the carcinomas were examined pathologically and classified according to the World Health Organization (WHO) classification [13], Union for International Cancer Control (UICC) TNM classification [14], and the Classification of Pancreatic Carcinoma of the Japan Pancreas Society [15]. Surgically resected specimens were fixed in 10% formalin and cut into serial 5-mm-thick slices, horizontally in the pancreas head, and by sagittal section in the pancreas body and tail. All of the sections were stained with hematoxylin and eosin (HE) for pathological examination.

### Immunohistochemistry

Immunohistochemistry was performed on formalin-fixed, paraffin-embedded (FFPE) tissue sections using the avidin–biotin complex method as described previously [16]. We used 4-μm-thick serial sections of each FFPE blocks with antibodies against the following: CD3 (PS1; 1:100) from Santa Cruz Biotechnology (Santa Cruz, CA, USA), CD4 (368; 1:100), and CD8 (4B11; 1:200) from Leica Microsystems (Newcastle-upon-Tyne, UK), and FOXP3 (42; 1:100) produced in-house [4]. Immunohistochemistry without the primary antibody was performed as a negative control.

### Quantitative evaluation of tumor-infiltrating T cell subsets

After immunohistochemistry, the microscopic images were imported as digital photo files using a NanoZoomer Digital Pathology System (Hamamatsu Photonics; Hamamatsu, Japan), and the density of the immunolabeled cells was analyzed using Tissue Studio image analysis software (Definiens, Munich, Germany) as described previously [17]. We confirmed all biopsy tissue was cancer tissue and manually selected total area of the biopsy specimen as the region of interest (ROI), in which the CD3-labeled T cells had infiltrated into the tumor. In each individual case, the same ROI was applied to all other immunostained images. The immunolabeled cells inside the ROI were automatically counted on the basis of staining intensity. In each analysis, we confirmed that the immunohistochemically positive lymphocytes were appropriately detected. The density of positive cells was calculated by dividing their number by the ROI area (cells/μm^2^). For survival and correlation analyses, patients were divided into two groups showing high and low cell infiltration, using the median value as a cut-off.

### RT-qPCR

Total RNA was extracted from fresh frozen samples of both tumor tissue and non-cancerous pancreas tissue, as described previously [18]. All samples were treated with rDNase during isolation, in accordance with the manufacturer's instructions. The quality of the extracted RNA was measured on an Agilent 2100 Bioanalyzer using an RNA 6000 Nano Kit (Agilent Technologies, Santa Clara, CA, USA); the rRNA ratio [28s/18s] and RNA integrity number (RIN) were 1.21 ± 0.18 and 7.2 ± 0.9 (average ± SD), respectively, as described previously [19]. RT-qPCR for target genes and non-target housekeeping control genes was performed on a 7500 Real-Time PCR System (Applied Biosystems, Foster City, CA) using FastStart Universal Probe Master (ROX) and probes from the Universal Probe Library (Roche Diagnostics Corp., Indianapolis, IN, USA), as described previously [18]. The sequences of the primers and the respective Universal Probe Library probes are given in Supplementary Table 3. The gene expression levels were normalized to the *ACTB* gene.

### Statistical analyses

Comparisons of qualitative variables were performed using the *χ*^2^ test or Fisher's exact test. The postoperative overall survival (OS) and disease-free survival (DFS) rates were calculated by the Kaplan–Meier method. Univariate analysis was performed for prognostic factors using the log-rank test. The factors found to be significant by univariate analysis were subjected to multivariate analysis using the Cox proportional hazards model (backward elimination method). Given that TILs are heterogeneously distributed within cancer tissues, we evaluated the number of times biopsy tissues should be collected in order to generate reliable data in TIL analysis by examining intra-rater reliability using an intraclass correlation, Case 1 (alternatively, one-way classification) model [20, 21]. We calculated the intraclass correlation coefficient (ICC) (1,1) for each variable based on the data of ten biopsies obtained from the identical cancer tissue (total of 17 cases). We estimated reliability levels in each biopsy frequency based on ICC (1, k). Differences at *P* < 0.05 were considered statistically significant. Statistical analyses were performed with the StatView-J 5.0 software (Abacus Concepts, Berkeley, CA, USA) and BellCurve for Excel (Social Survey Research Information Co., Ltd., Tokyo, Japan).

## SUPPLEMENTARY MATERIALS FIGURES AND TABLES


