# Supplementary material for: Reliable evaluation of tumor-infiltrating lymphocytes in pancreatic cancer tissue biopsies
Source: Oncotarget. 2019 Feb 1;10(10):1149–59. doi: 10.18632/oncotarget.26646 (PMC6383689; doi:10.18632/oncotarget.26646)
Supplement: Supplementary file 1 [file oncotarget-10-1149-s001.pdf]

Reliable evaluation of tumor-infiltrating lymphocytes in pancreatic cancer tissue biopsies

SUPPLEMENTARY MATERIALS

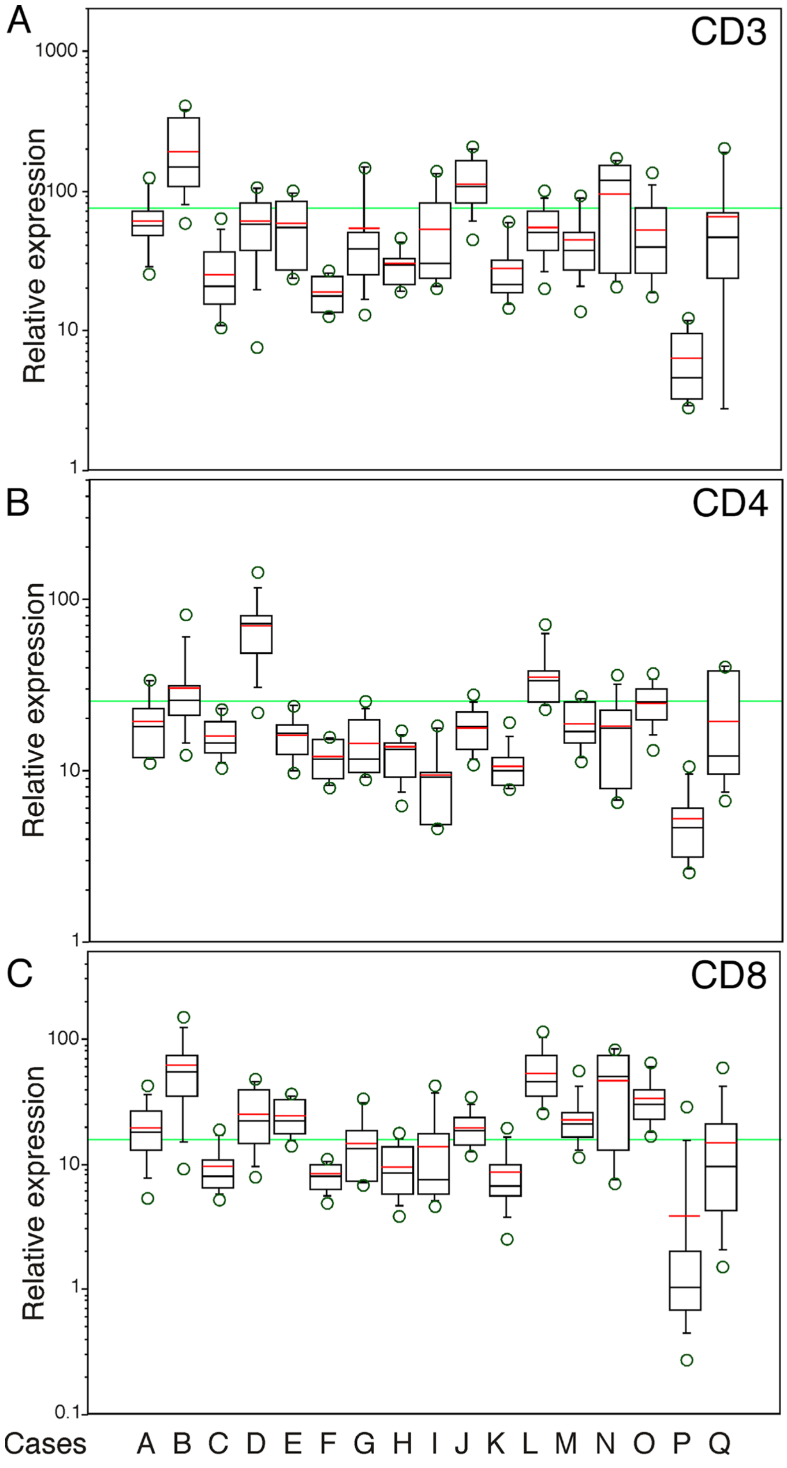

Supplementary Figure 1: Box plots for expression of *CD3* (**A**), *CD4* (**B**), and *CD8* (**C**) genes in PDAC biopsy tissues. The red line indicates the gene expression level in the corresponding surgically-resected tissue sample. The green line indicates the gene expression cut-off value set at the median of 241 cases of surgically-resected PDAC.

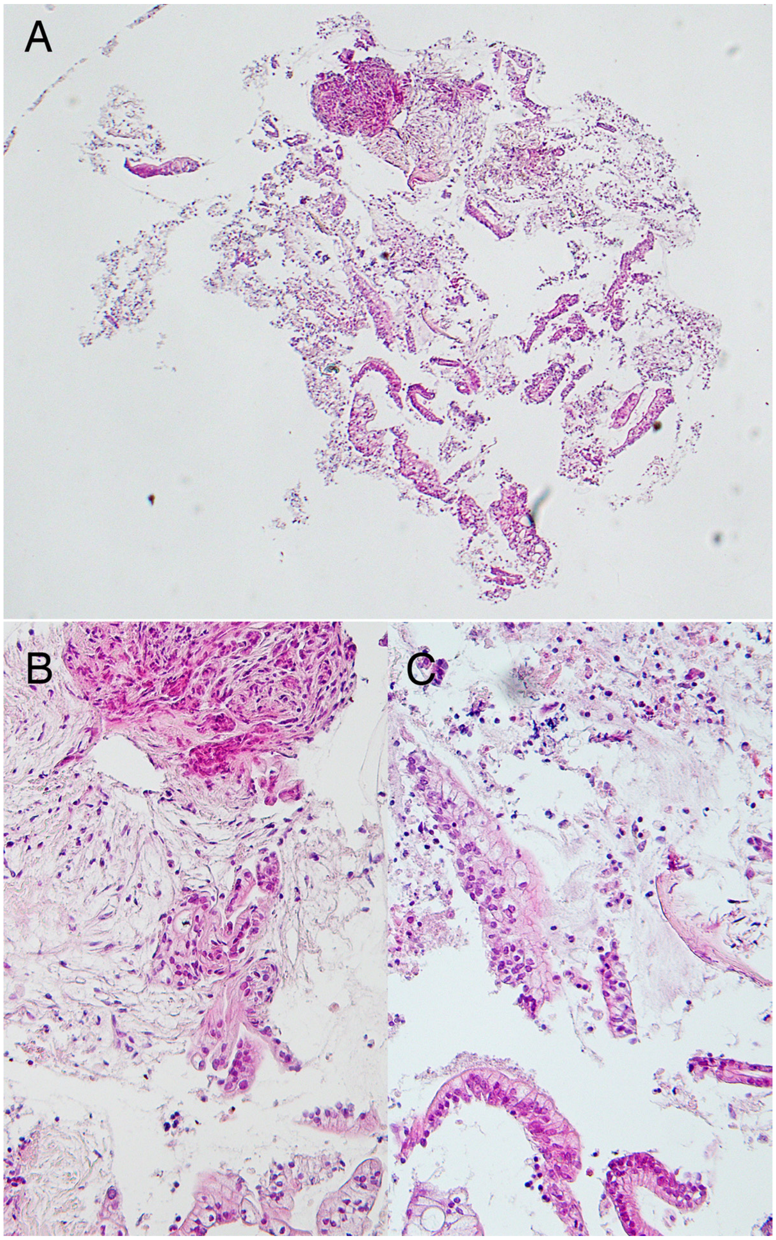

Supplementary Figure 2: Representative biopsied materials stained with hematoxylin and eosin. Only very small-sized and fragmented tissues and cells are obtained. Very low magnification (**A**) and low magnification (**B, C**).

Supplementary Table 1: Median survival time, one-, two-, and five-year survival rate

Variables	Group	Overall survival				Disease-free survival			
		Median survival time[a] (months)	Survival rate (%)			Median survival time[a] (months)	Survival rate (%)		
			1-yr	2-yr	5-yr		1-yr	2-yr	5-yr
Expression of *CD3*	Low	24.3 ± 4.4	100	53.6 ± 20.0	53.6 ± 20.0	24.3 ± 4.4	100	53.6 ± 20.0	53.6 ± 20.0
	High	NA	100	83.3 ± 15.2	83.3 ± 15.2	NA	100	83.3 ± 15.2	83.3 ± 15.2
Expression of *CD4*	Low	24.3 ± 4.0	100	51.4 ± 20.4	51.4 ± 20.4	24.3 ± 4.0	100	51.4 ± 20.4	51.4 ± 20.4
	High	NA	100	85.7 ± 13.2	85.7 ± 13.2	NA	100	85.7 ± 13.2	85.7 ± 13.2
Expression of *CD8*	Low	24.3 ±3.8	100	60.0 ± 21.9	60.0 ± 21.9	24.3 ±3.8	100	60.0 ± 21.9	60.0 ± 21.9
	High	NA	100	75.0 ± 15.3	75.0 ± 15.3	NA	100	75.0 ± 15.3	75.0 ± 15.3
Expression of *FOXP3*	Low	24.3 ± 4.5	100	58.3 ± 18.6	58.3 ± 18.6	24.3 ± 4.5	100	58.3 ± 18.6	58.3 ± 18.6
	High	NA	100	83.3 ± 15.2	83.3 ± 15.2	NA	100	83.3 ± 15.2	83.3 ± 15.2
Ratio of *FOXP3/CD4*	Low	NA	100	72.9 ± 16.5	72.9 ± 16.5	NA	100	72.9 ± 16.5	72.9 ± 16.5
	High	24.3 ± 3.7	100	62.5 ± 21.4	62.5 ± 21.4	24.3 ± 3.7	100	62.5 ± 21.4	62.5 ± 21.4

Abbreviation: NA: not available, [a]median ± standard error.

Supplementary Table 2: Clinicopathological characters of PDAC using biopsy model analysis

Age, years		
	<65	8
	≥65	9
Gender		
	Male	9
	Female	8
Pathologic tumor status		
	T1a/1b/1c	0/0/0
	T2	14
	T3	3
	T4	0
Pathologic node status		
	N0	6
	N1	5
	N2	6
Pathologic metastasis status		
	M0	15
	M1	2
Stage		
	IA	0
	IB	5
	IIA	1
	IIB	5
	III	4
	IV	2
Tumor grade		
	Grade 1	0
	Grade 2	13
	Grade 3	4
Nerve plexus invasion[a]		
	Absence	4
	Presence	13
Lymphatic invasion[a]		
	0, 1	1
	2, 3	16
Venous invasion[a]		
	0, 1	1
	2, 3	16
Intrapancreatic neural invasion[a]		
	0, 1	3
	2, 3	13
Tumor margin status		
	Negative	13
	Positive	4
Adjuvant chemotherapy		
	Negative	3
	Positive	14
Total		17

[a]Classified according to the classification of pancreatic carcinoma of Japan Pancreas Society.

Supplementary Table 3: Primer sequences and Universal Probe Library probes for quantitive RT-PCR

Name of genes	Universal Probe Library Probe	Left primer	Right primer
ACTB	#64	CCAACCgCgAgAAgATgA	TCCATCACgATgCCAgTg
CD3	#58	TCCTTgCTgTTggggTCTAC	CAgAgTCTgCTTgTCTgAAgCTC
CD4	#18	ggCAgTgTCTgCTgAgTgAC	gACCATgTgggCAgAACCT
CD8	#51	TCATggCCTTACCAgTgACC	AggTTCCAggTCCgATCC
FOXP3	#20	gAgAAgCTgAgTgCCATgC	AgCCCTTgTCggATgATg
